# Analysis of the fecal microbiota of fast- and slow-growing rainbow trout (*Oncorhynchus mykiss*)

**DOI:** 10.1186/s12864-019-6175-2

**Published:** 2019-10-29

**Authors:** Pratima Chapagain, Brock Arivett, Beth M. Cleveland, Donald M. Walker, Mohamed Salem

**Affiliations:** 1 Department of Biology and Molecular Biosciences Program, Middle Tennessee State University, Murfreesboro, TN 37132 USA; 2 Department of Chemistry, Middle Tennessee State University, Murfreesboro, TN 37132 USA; 3 National Center for Cool and Cold-Water Aquaculture, ARS-USDA, Kearneysville, WV 25430 USA; 4 Department of Animal and Avian Sciences, University of Maryland, College Park, MD 20742 USA

**Keywords:** Aquaculture, Trout, Gut, Microbiota, DNA-isolation, Breeding

## Abstract

**Background:**

Diverse microbial communities colonizing the intestine of fish contribute to their growth, digestion, nutrition, and immune function. We hypothesized that fecal samples representing the gut microbiota of rainbow trout could be associated with differential growth rates observed in fish breeding programs. If true, harnessing the functionality of this microbiota can improve the profitability of aquaculture. The first objective of this study was to test this hypothesis if gut microbiota is associated with fish growth rate (body weight). Four full-sibling families were stocked in the same tank and fed an identical diet. Two fast-growing and two slow-growing fish were selected from each family for 16S rRNA microbiota profiling.

Microbiota diversity varies with different DNA extraction methods. The second objective of this study was to compare the effects of five commonly used DNA extraction methods on the microbiota profiling and to determine the most appropriate extraction method for this study. These methods were Promega-Maxwell, Phenol-chloroform, MO-BIO, Qiagen-Blood/Tissue, and Qiagen-Stool. Methods were compared according to DNA integrity, cost, feasibility and inter-sample variation based on non-metric multidimensional scaling ordination (nMDS) clusters.

**Results:**

Differences in DNA extraction methods resulted in significant variation in the identification of bacteria that compose the gut microbiota. Promega-Maxwell had the lowest inter-sample variation and was therefore used for the subsequent analyses. Beta diversity of the bacterial communities showed significant variation between breeding families but not between the fast- and slow-growing fish. However, an indicator analysis determined that cellulose, amylose degrading and amino acid fermenting bacteria (*Clostridium*, *Leptotrichia,* and *Peptostreptococcus*) are indicator taxa of the fast-growing fish. In contrary, pathogenic bacteria (*Corynebacterium* and *Paeniclostridium*) were identified as indicator taxa for the slow-growing fish.

**Conclusion:**

DNA extraction methodology should be carefully considered for accurate profiling of the gut microbiota. Although the microbiota was not significantly different between the fast- and slow-growing fish groups, some bacterial taxa with functional implications were indicative of fish growth rate. Further studies are warranted to explore how bacteria are transmitted and potential usage of the indicator bacteria of fast-growing fish for development of probiotics that may improve fish health and growth.

## Introduction

The efficiency and profitability of industrial aquaculture depend in part on the growth rate of farmed fishes. Growth in farmed fishes is a complex process that is directly dependent on host genetics, food quality and availability, and environmental conditions [1]. Selective breeding is one strategy that can be used to improve important phenotypic traits and help in understanding the genetic architecture and the role of molecular factors causing genetic variation among different fish [2]. Family-based selection procedures have been undertaken by the United States Department of Agriculture (USDA), National Center for Cool and Cold-Water Aquaculture (NCCCWA) to improve growth rate, fillet quality and disease resistance of rainbow trout. A growth-selected line was developed starting in 2002, and since then yielded a genetic gain of approximately 10% in improved growth performance per generation [3].

Microorganisms may also contribute to the productivity of farmed fishes. Microorganisms making up the fish microbiota reside on the fish skin, gills, and gastrointestinal tract and likely play a crucial role in the growth rate, metabolism, and immunity of the fish host [4, 5]. While host genetics has a profound role in determining the gut microbiome of humans and other mammals, it is not well studied in fish [6–9]. On the other hand, feed and water in which fish are reared have vital roles in shaping the gut microbiome. For example, plant and animal-based meal can widely alter the composition of the host microbiota since fish acquire their microbiota from the first-feed they eat [10–12]. Sharp et al. reported that microbiota of the marine species can be directly inherited from ancestors and passed from generation to generation [13]. The gut, in particular, features a diverse microbiota contributing to the weight gain, immune development, pathogen inhibition, and various metabolic activities of the hosts [14]. Resident gut microbes are beneficial for hosts either by inhibiting pathogenic bacteria with dedicated toxins or by secreting enzymes that breakdown indigestible polysaccharides in host gut to simple monosaccharides and short-chain fatty acids [15]. Gut microbes can supply compounds such as vitamin B and K to host which may improve the host energy metabolism [16].

An accurate census of bacteria from fish may allow investigation of the positive effects of the microbiota. However, profiling of the gut microbiome is directly influenced by many factors including the experimental design, sample collection, and processing. DNA extraction is particularly important since microbiome analysis requires adequate quality and quantity of DNA isolated for an accurate representation of the host-microbiome [17]. Many protocols have been commercialized for DNA extraction and previous reports demonstrate that microbiome diversity varies with different DNA extraction methods [18]. It is difficult to determine the most appropriate extraction method for the downstream microbiome analysis of a particular species. Each method has its own merits and drawbacks; for example, standardized kits are typically designed for ease of use and efficiency, but a more labor-intensive method such as Phenol-chloroform extraction, despite its risk of inconsistency or contamination, can potentially produce a higher yield with better quality if performed by an experienced researcher.

In this study, we investigated how the gut microbiota of rainbow trout correlates with differential growth rates. Therefore, one objective of this research was to characterize the gut microbiota of rainbow trout using high-throughput DNA sequencing. In order to achieve this objective, we considered the effect that DNA extraction methodologies play in the characterization of different microbial communities in the gut of rainbow trout. The specific objectives of our study were to determine differences in community structure of the gut microbiota between fast- and slow-growing rainbow trout and to determine if genetics plays a role in determining the gut microbiota profile. Our results highlight differences of the gut microbiota between fish family and the bacterial taxa indicative of fast- and slow-growing rainbow trout.

## Results

### Comparison of different DNA extraction methods

**Fig. 1:**
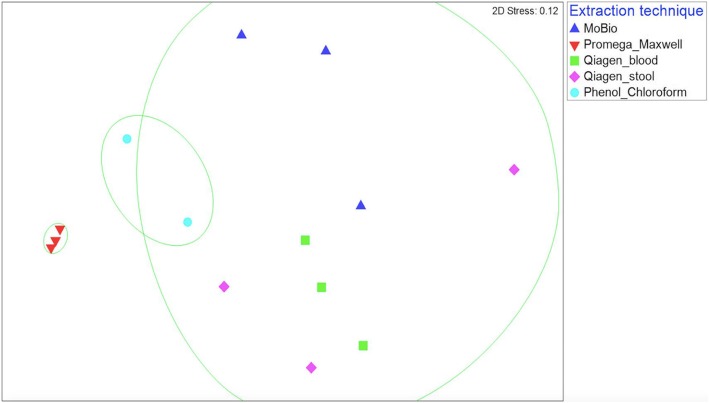
nMDS representation of three replicate pooled samples using 5 different extraction methods (stress value = 0.12). Each extraction method is significantly different (*p* < 0.05). SIMPROF analysis tested for significant distinct clusters. One of the phenol-chloroform samples did not pass the QC and was excluded from the analysis

To test if profiling of the gut microbiota is directly influenced by the DNA extraction method, three replicate pools of the fish fecal samples were sequenced and analyzed using five different extraction methods. Within non-metric dimensional scaling ordination plots, the three-replicate samples extracted with Promega clustered tightly, whereas, replicate samples of the four other extraction methods were relatively more heterogeneous (Fig. 1). PERMANOVA confirmed that the microbial population differs on using different DNA extraction method (F_4,13_ = 2.4234, *p* < 0.05, R^2^ = 51%).

**Fig. 2:**
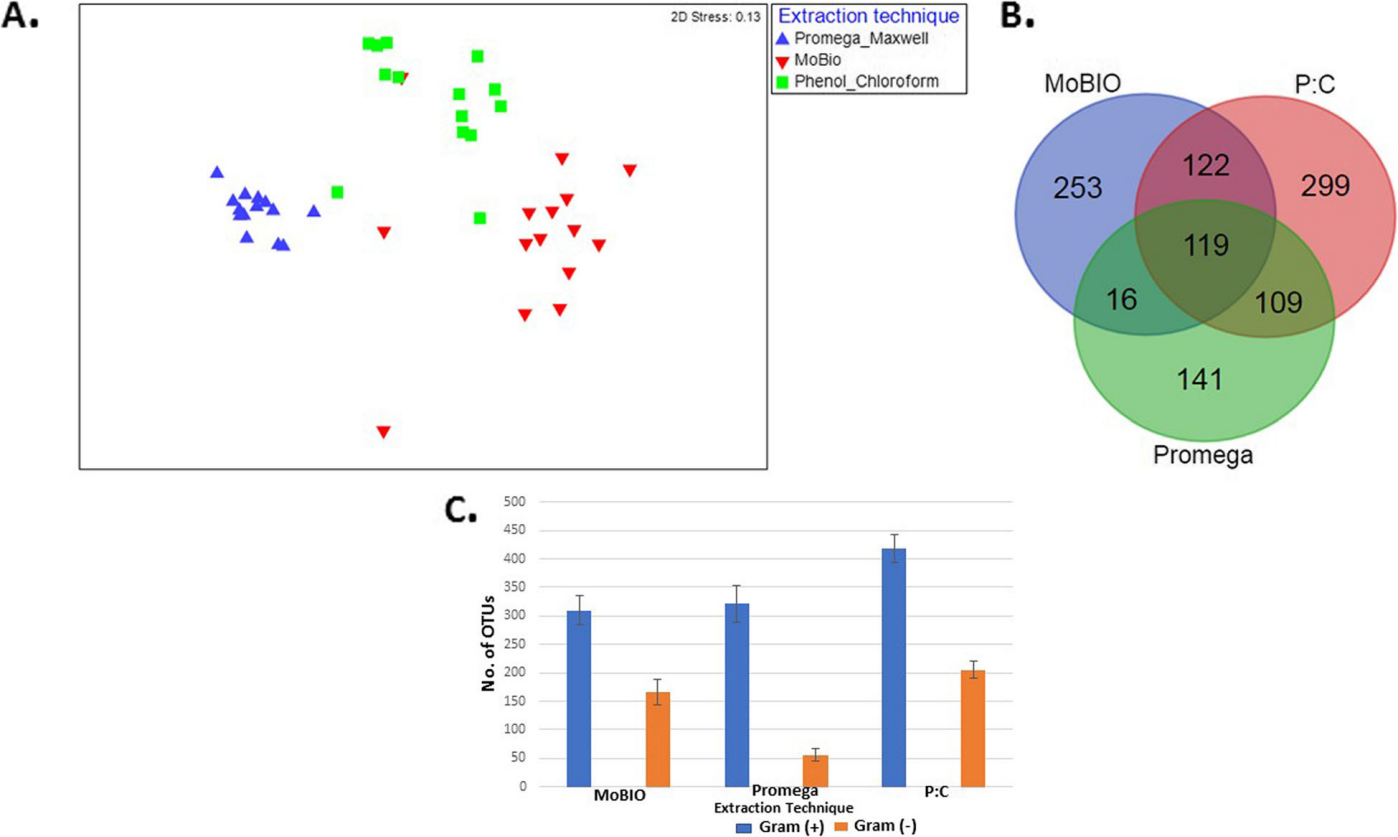
**a**) nMDS representation of the fecal samples using three different extraction methods. Samples were clustered on the basis of Bray-Curtis distance matrices (stress value = 0.13). **b**) Venn Diagram depicting the common and unique OTUs in three different extraction methods, P:C indicates phenol-chloroform **c**) Abundance of Gram-positive and Gram-negative bacteria on rainbow trout gut using three different extraction methods. The error bar indicates the standard deviation

To further investigate the effects of DNA extraction methodology on microbiota profiling, three different methods were chosen for microbiota sequencing from the individual (non-pooled) biological replicate fecal samples of all available fish in the study. PERMANOVA results confirmed the significant effect of extraction technique on predicting microbial communities (Fig. 2 a; F_2, 42_ = 10.467, *p* < 0.05, R^2^ = 34%). Comparative analysis of the three extraction methods revealed that Phenol-chloroform had the highest OTU richness with 649 OTUs. A total of 119 OTUs overlapped between all three DNA isolation methods (Fig. 2b). Comparing the abundance of the Gram-positive and Gram-negative bacteria, it was clear that the abundance of the Gram-positive is higher than that of the Gram-negative in all three DNA extraction techniques (Fig. 2c) with the Promega kit being the highest. The SIMPROF test for statistically significant cluster and it showed that the Promega method had 95% similarity within the individual samples forming the tightest cluster (*p* < 0.05).

Beside heterogeneity and abundance biases, other factors including yield, integrity, time durations for sample processing, the amount of hazardous waste liberated were also considered during extraction comparison. Phenol-chloroform gave the highest yield, but it is tedious, time-consuming, requires individual handling and released more hazardous waste whereas, Promega is a semi-automated method, easy to perform in large-scale production, and showed the least inter-sample variation among the replicate samples, results in release of least hazardous waste as shown in (Table 1). We decided to choose Promega for our downstream analysis of the fecal microbiota.

**Table 1:** Comparison of five different DNA extraction methods for microbiota analysis on the basis of cost, concentration, and the time duration for sample processing

Extraction Kit	Manufacturer	Principle	Bead Beating	Concentration (ng/μl)	A260/230	Cost per sample	Time duration	Hazardous waste
Power Soil	MoBio	Manual	Yes	6.49 ± 9.09	1.78 ± 0.18	$6.48	6 h	Moderate
Maxwell	Promega	Automated	Yes	28.76 ± 12.44	1.72 ± 0.17	$7.40	1.5 h	Least
Phenol:Chloroform	Sigma	Manual	No	257.1 ± 285.0	1.73 ± 0.08	$4.50	2 days	High
Qiagen_Stool	Qiagen	Manual	No	25.1 ± 10.07	1.92 ± 0.16	$5.60	5 h	Less
Qiagen_Blood/Tissue	Qiagen	Manual	No	35.2 ± 2.7	1.72 ± 0.01	$4.20	5 h	Less

### Mean weight difference between fast and slow-growing fish

**Fig. 3:**
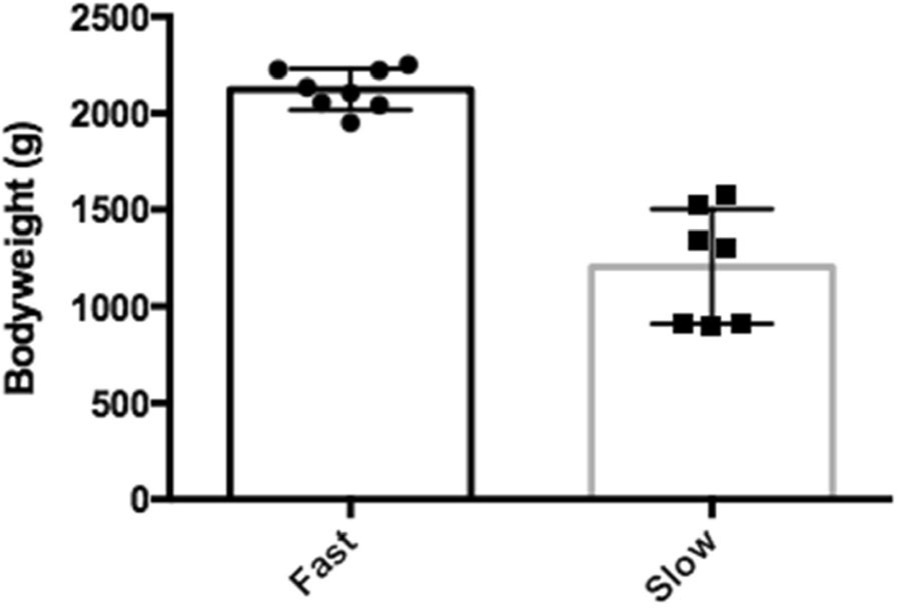
Significant difference in the mean weight of the fast-growing versus slow-growing fish used in the study. The statistical significance of the rank body mass between the two groups was tested by a one-way Mann-Whitney U test (p < 0.05). The error bars indicate standard deviation

The mean weight of the fast-growing fish was 2123.9 ± 105.57 g, whereas, the mean weight of the slow-growing fish was 988.6 ± 297.65 g. The mass of the fast-growing fish was significantly greater than that of the slow-growing fish when compared using one-way Mann-Whitney U test (*p* < 0.05) as shown in Fig. 3.

### Gut microbiota analysis of fast- and slow-growing fish

**Fig. 4:**
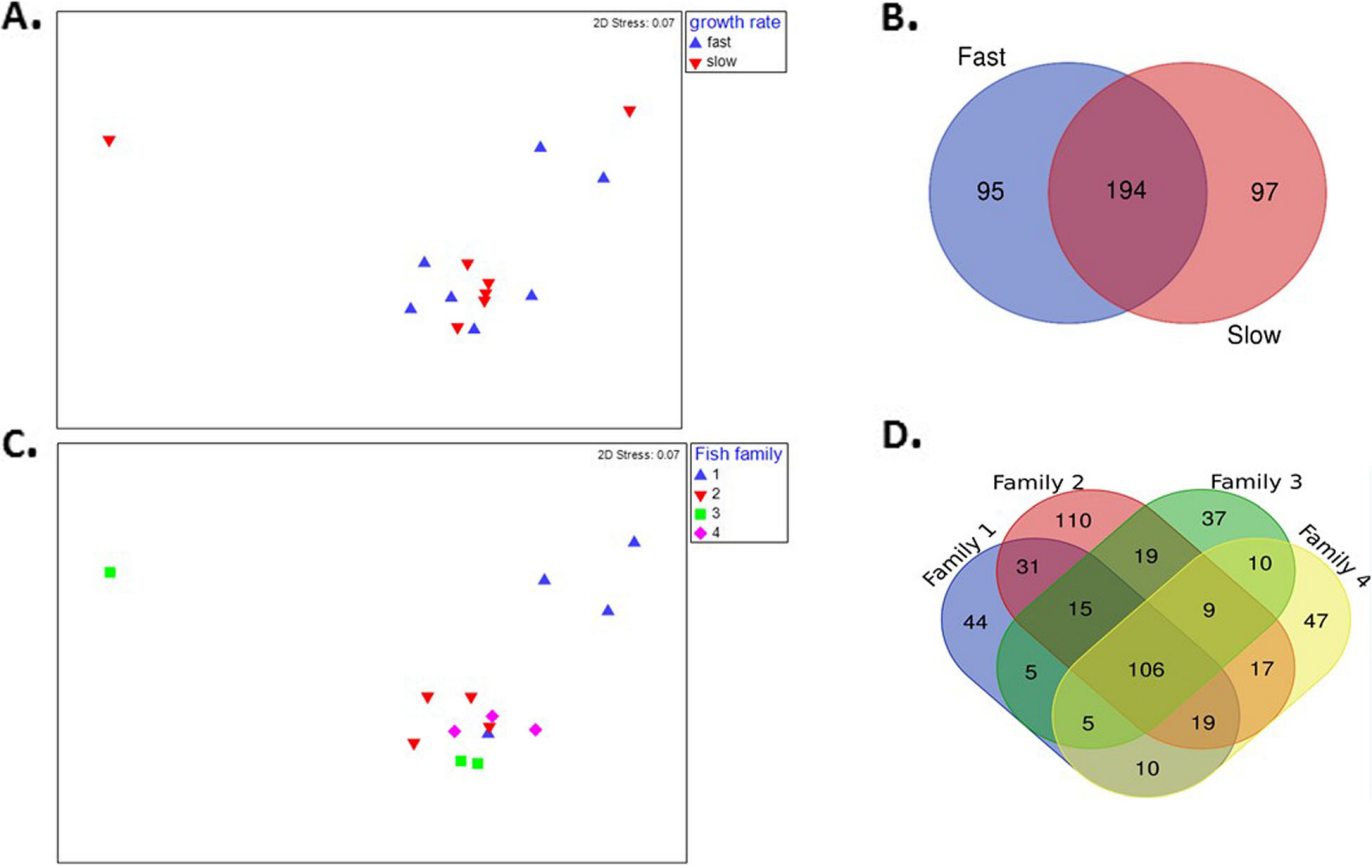
**a**) nMDS representation of the fast- and slow-growing fish using Promega extraction method (stress value = 0.07). **b**) Venn-diagram depicting the common and unique OTUs in fast-growing and slow-growing rainbow trout **c**) nMDS representation of the fish family on the basis of dissimilarity matrices (stress value = 0.07). Most of the samples from family 1 were clustered apart from families 2, 3, and 4. **d**) Venn representation of the common and unique OTUs among four different families

Our analysis of microbial diversity based on alpha diversity in the fast-growing and slow-growing fish fecal samples using inverse Simpson indices indicated no significant differences between fast and slow-growing fish (*p* > 0.05, data not shown). Moreover, both nMDS ordination and PERMANOVA results indicated that the microbial communities did not significantly differ between the fish of different growth rates (p > 0.05, Fig. 4a). Both fast- and slow-growing fish possessed unique sets of OTUs and overlapping taxa (Fig. 4b). However, an indicator analysis predicted that 10 OTUs were found as indicative of the growth rate (Table 2, *p* < 0.05). All fast-growing indicator taxa belonged to phylum Firmicutes, including *genera Clostridium, Sellimonas, Leptotrichia, Tepidimicrobium, Peptostreptococcus* and Lachnospiraceae_unclassified whereas, the slow-growing indicator taxa belonged to phylum Actinobacteria and Firmicutes with genera *Corynebacterium and Paeniclostridium* (Table 2).

**Table 2:** Indicator analysis of the taxa for growth rate using Mothur

Growth	Phylum	Class	Order	Family	Genus	Abundance	Indicator Value	*P*-value
Fast	Firmicutes	Clostridia	Clostridiales	Clostridiaceae_1	Clostridium_sensu_stricto_1	1589	86	< 0.001
	Firmicutes	Clostridia	Clostridiales	Lachnospiraceae	Sellimonas	1265	66	0.03
	Fusobacteria	Fusobacteriia	Fusobacteriales	Leptotrichiaceae	Leptotrichia	940	75	0.03
	Firmicutes	Clostridia	Clostridiales	Clostridiaceae_1	Clostridium_sensu_stricto_18	761	78	0.04
	Firmicutes	Clostridia	Clostridiales	Family_XI	Tepidimicrobium	456	77	0.03
	Firmicutes	Bacilli	Bacillales	Planococcaceae	Planococcaceae_unclassified	388	79	0.01
	Firmicutes	Clostridia	Clostridiales	Lachnospiraceae	Lachnospiraceae_unclassified	357	78	0.02
	Firmicutes	Clostridia	Clostridiales	Peptostreptococcaceae	Peptostreptococcus	139	80	0.01
Slow	Actinobacteria	Actinobacteria	Corynebacteriales	Corynebacteriaceae	Corynebacterium_1	10,033	74.07	0.01
	Firmicutes	Clostridia	Clostridiales	Peptostreptococcaceae	Paeniclostridium	958	65	0.04

*p* ≤ 0.05 indicates the significant taxa to act as indicator of the fast-growing or slow-growing fish

In addition, PERMANOVA results indicated differences in the microbiota among the fish families (F_3,13_ = 2.1673, *p* < 0.05, R^2^ = 39%) (Fig. 4c). The Venn-representation depicted 106 OTUs shared among all the families with family 2 having the most unique OTUs (Fig. 4d). An indicator analysis of each fish family predicted that six OTUs belonging to phylum Actinobacteria and Firmicutes including genera *Truperella, Kocuria, Lactobacillus, Lactococcus* were identified as indicative of family 1. Three OTUs belonging to phylum Fusobacteria, Firmicutes including genera *Fusobacterium and Peptostreptococcus* were indicator taxa for family 2. And one OTUs belonging to phylum Proteobacteria including genus *Pseudomonas* was indicator taxa for family 4 (Table 3, *p* < 0.05). The overall taxa information of the fecal samples has been included in Additional file 1.

**Table 3:** Indicator analysis of the taxa for fish families using Mothur

Fish Family	Phylum	Class	Order	Family	Genus	Abundance	Indicator value	*p*-value
1	Actinobacteria	Actinobacteria	Actinomycetales	Actinomycetaceae	Trueperella	9007	53.15	0.02
	Actinobacteria	Actinobacteria	Micrococcales	Micrococcaceae	Kocuria	5226	57.95	0.007
	Firmicutes	Bacilli	Lactobacillales	Lactobacillaceae	Lactobacillus	1233	68.78	0.02
	Firmicutes	Clostridia	Clostridiales	Ruminococcaceae	Ruminococcaceae_UCG-014	615	65.49	0.03
	Firmicutes	Bacilli	Lactobacillales	Streptococcaceae	Lactococcus	589	73.38	0.015
	Actinobacteria	Actinobacteria	Propionibacteriales	Propionibacteriaceae	Propionibacteriaceae	134	52.7	0.02
	Fusobacteria	Fusobacteriia	Fusobacteriales	Fusobacteriaceae	Fusobacterium	1048	61.53	0.03
2	Firmicutes	Clostridia	Clostridiales	Peptostreptococcaceae	Peptostreptococcus	110	65.57	0.02
	Firmicutes	Clostridia	Clostridiales	Family_XIII	Family_XIII_unclassified	86	63.15	0.03
	Bacteroidetes	Bacteroidia	Bacteroidales	Bacteroidales_unclassified	Bacteroidales_unclassified	12,125	99.49	0.04
3	Firmicutes	Bacilli	Bacillales	Paenibacillaceae	Paenibacillus	360	70.31	0.019
	Actinobacteria	Coriobacteriia	Coriobacteriales	Atopobiaceae	Atopobiaceae_unclassified	196	63.414	0.01
4	Proteobacteria	Gammaproteobacteria	Pseudomonadales	Pseudomonadaceae	Pseudomonas	5265	76.19	0.01

*p* ≤ 0.05 indicates the significant indicator taxa for each fish family

Because the Phenol-chloroform yielded higher OTUs, despite the higher intersample variation among the replicates, as a curiosity, we ran the nMDS ordination and PERMANOVA analyses using the Phenol-chloroform extraction method. The results also indicated no significant differences among the growth rate (p < 0.05) of fish with significant differences among the families (p < 0.05) and alpha diversity analysis using inverse Simpson index also showed insignificant results (*p* > 0.05). These results resemble those obtained by the Promega extraction method.

## Discussion

In this study, the DNA extraction methodology comparison was performed to optimize the extraction methodology and apply this to the comparison of fast- and slow-growing fish gut microbiota. Five different extraction techniques, including bead beating and semi-automated methods, were examined. The effects of the DNA extraction methods were assessed on the basis of the DNA quantity, quality and the inter-sample variation in microbial communities between replicates. The concentration and the quality of the DNA varied significantly between the DNA extraction techniques. The MOBIO, Qiagen Blood/Tissue and Qiagen Stool gave relatively low yield, whereas Promega Maxwell kit that uses automated method resulted in a higher yield in comparison to the other kits which is consistent with previous reports [19]. In comparison, Phenol-chloroform, being a robust method, uses a stringent lysis step and produced the highest DNA yield and highest microbial diversity. This is likely due to the Phenol-chloroform method being able to effectively lyse the cell walls of both the Gram-positive and Gram-negative bacteria. However, the Phenol-chloroform method resulted in higher inter-sample variation, is the most labor-intensive, and produces more hazardous waste when compared to the Promega method. It has been proven that the bead-beating methods result in the identification of greater microbial diversity than non-beating methods [20]. MOBIO method, involves bead beating to physically lyse cell wall of bacteria, increased the number of the microbial species identified but showed relatively high inter-sample variation among replicates. Promega Maxwell, a semi-automated method, also includes bead-beating steps, however, yielded a higher abundance of Gram-positive bacteria, perhaps, due to addition of lysozyme enzymes, which induces lysis of the Gram-positive bacterial cell wall. The Promega method showed the least inter-sample variation among technical replicates. Similar is the case with Qiagen-stool, Qiagen-Blood/Tissue kits since both methods gave sufficient yield and integrity but resulted in higher inter-sample variation among replicates.

We found that specific taxa were indicators of the fish growth rate and fish breeding family. The indicator taxa associated with slow growth rate seem to be harmful/pathogenic bacteria, whereas the indicator taxa of fast-growing fish seem to have a mutually beneficial relationship with the host. *Corynebacterium* and *Paeniclostridium* which are known pathogens [21] were more prevalent in slow-growing fish. The toxins produced by these bacteria cause swelling and abdominal discomfort due to fluid accumulation and sometimes also lead to the development of circumscribed lesions and lethargic behavior [22]. Families *Lachnospiraceae*, *Leptotrichiaceae*, *Planococcaceae*, and *Peptostreptococcaceae* belonging to the phylum Firmicutes were indicator taxa for the fast-growing fish in this study. Firmicutes impact fatty acid absorption and lipid metabolism, thus expected to affect body weight in the host [23–25]. A study done in Zebrafish explained the contribution of Firmicutes in stimulating the host metabolism and increasing the bioavailability of fatty acids by modifying bile salts [26]. Bacteria belonging to class *Lachnospiraceae* reside in the digestive tract, produce butyric acid, aid in amino acid fermentation, protein digestion, absorption of fatty acids, were associated with weight gain and prevention of different diseases due to microbial and host epithelial cell growth [27, 28]. On the other hand, bacteria like *Sellimonas*, *Clostridium*, *Peptostreptococcus* in fast-growing fish can take part in fermentation of different amino acids, lactates and sugars [29]. *Clostridium* is more likely to produce cellulase enzyme and result in degradation of the cellulolytic fibers. The most widely prevalent and statistically significant indicator taxa of the fast-growing fish, *Peptostreptococcus* and *Clostridium*, are more likely to be involved in amino acid fermentation that ultimately leads to amino acid absorption in host gut. *Leptorichia*, the most abundant taxa in the gut of all the fast-growing fish are cellulose-degrading bacteria; therefore, amylase and cellulase activities are expected to be more prominent in the host inhabiting these bacteria [30]. Similarly, the class *Enterobacteriaceae* was found to be a significantly abundant taxonomical class in most of the fast-growing fish*. E. coli* belonging to class *Enterobacteriaceae* has proven to be associated with weight gain in human infants [31].

Although most of the microbiota were shared among the fish families, some unique taxa were characteristic for each family, which suggests that genetics is a contributing factor affecting the gut microbiota. Unique taxa for fish family 1 included *Trueperiolla, Kocuria, Lactobacillus*, *Lactococcus,* and *Propionibacteriaceae. Kocuria* has been reported to induce the protective immune system in rainbow trout by inhibiting pathogenic bacteria like Vibrio [32]*. Lactobacillus* has been found to inhibit the pathogens and, therefore, used as preservatives for food storage since they can induce the barrier function in the host epithelium against pathogens [33]. Also, bacteria belonging to family *Propionibacteriaceae* produce microbial metabolites such as short-chain fatty acids during glucose fermentation [34]. The bacteria belonging to this family also produce enzymes for fatty acid degradation that may help in the breakdown of food and produce valuable nutrients and energy [29, 35–37]. Similarly, *Fusobacterium*, an indicator taxon of fish family 2 produces butyrate which supplies energy, enhances mucus production and induces anti-inflammatory properties in the host [38]. Fish family 3 showed a higher abundance of phylum Bacteroidales with unclassified family and genus. *Bacteriodetes* belonging to this phylum produces inhibitory substances like bacteriocin which initiates pathogenic bacterial cell lysis or growth inhibition [35]. *Pseudomonas*, an indicator taxon of family 4 has been identified as the gut microbiota that aid in digestion [10]. Differences in microbiota among the families suggest that host genetics may create a genetic background that promotes the specific selection of microbiota from the environment. However, it should also be acknowledged that early periods of development, before fish comingled for the grow-out period, occurred in different tanks specific to each family. Although all four tanks were positioned sequentially, utilized the same water source (inlets came originated from the same pipe), and consumed identical feed, it is unknown if the microbial communities within each tank differed and, if so, how they could have persisted through the subsequent 12-month grow-out period. It is also unknown if there is vertical microbiota transmission from the parents to progeny or if maternal fecal contamination of eggs during manual egg stripping contributes to the offspring microbiota. Further research is needed to validate familial differences and determine the contribution of genetic and environmental factors to development of the gut microbiota.

## Conclusion

This study showed that DNA extraction methodology should be taken into account for accurate profiling of the gut microbiome. Some bacterial taxa were found to be significantly different between fish families, perhaps due to host genetics, unique early rearing environments, or vertical microbiota transmission. Although population-level microbiota differences were not found to be significantly associated with the fish growth rate, several indicator taxa were determined in the fast- and slow-growing fish. For future studies, some of these taxa can be investigated for potential use as probiotics to improve the gut microbiota of rainbow trout. Overall, our study investigated the gut-passing microbiota using fecal samples, which may not represent the mucosal microbiota.

## Methods

### Fish population

Fecal samples were collected from 15 fish representing four different genetic families. The parents of these families originated from a growth-selected line at NCCCWA (year class 2014) that was previously described [3, 39]. Fish families were produced and reared at NCCCWA until ~ 18 months post-hatch. Briefly, full-sibling families were produced from single-sire × single-dam mating events. All sires were siblings from a single-family while dams exhibited low relatedness (coefficient of relatedness < 0.16). Eggs were reared in spring water, and water temperatures were manipulated between approximately 7–13 °C to synchronize hatch times. Each family was reared separately from hatch through approximately 20 g (7 months post-hatch) when 15 fish per family were uniquely tagged by inserting a passive integrated transponder (Avid Identification Systems Inc., Norco, CA) into the peritoneal cavity. Tagged fish were comingled for the remainder of the grow-out period. Fish were fed a commercial fishmeal-based diet (42% protein, 16% fat, Ziegler Bros Inc., Gardners, PA) using automatic feeders (Arvotec, Huutokoski, Finland). Feed was provided at or just below satiation for the entire grow-out period. This study includes four families with high variance in adult body weight. From each family, four fish were selected, two that were considered fast-growing (> 1952 g) and two that were slow-growing (< 1572 g). Of the 16 fish selected for sampling, one slow-growing fish from family two exhibited morphological signs of disease during sample collection and was excluded from analysis, reducing the total number of samples to 15.

### Sample collection

To characterize the gut microbiota, samples were collected from fish feces. For fecal sampling, fish were anesthetized with MS-222 (tricane methane sulfonate) at a concentration of 150 mg m/L (Tricaine-S, Western Chemical, Ferndale, WA) and then manually stripped to collect the fecal samples in sterile Eppendorf tubes (Eppendorf, Hauppauge, NY). All samples were stored at − 80 °C until DNA extraction. At the end of the experiment, fish were euthanized with an overdose of MS-222 at a concentration of 300 mg/L.

### DNA isolation and sequencing

**Fig. 5:**
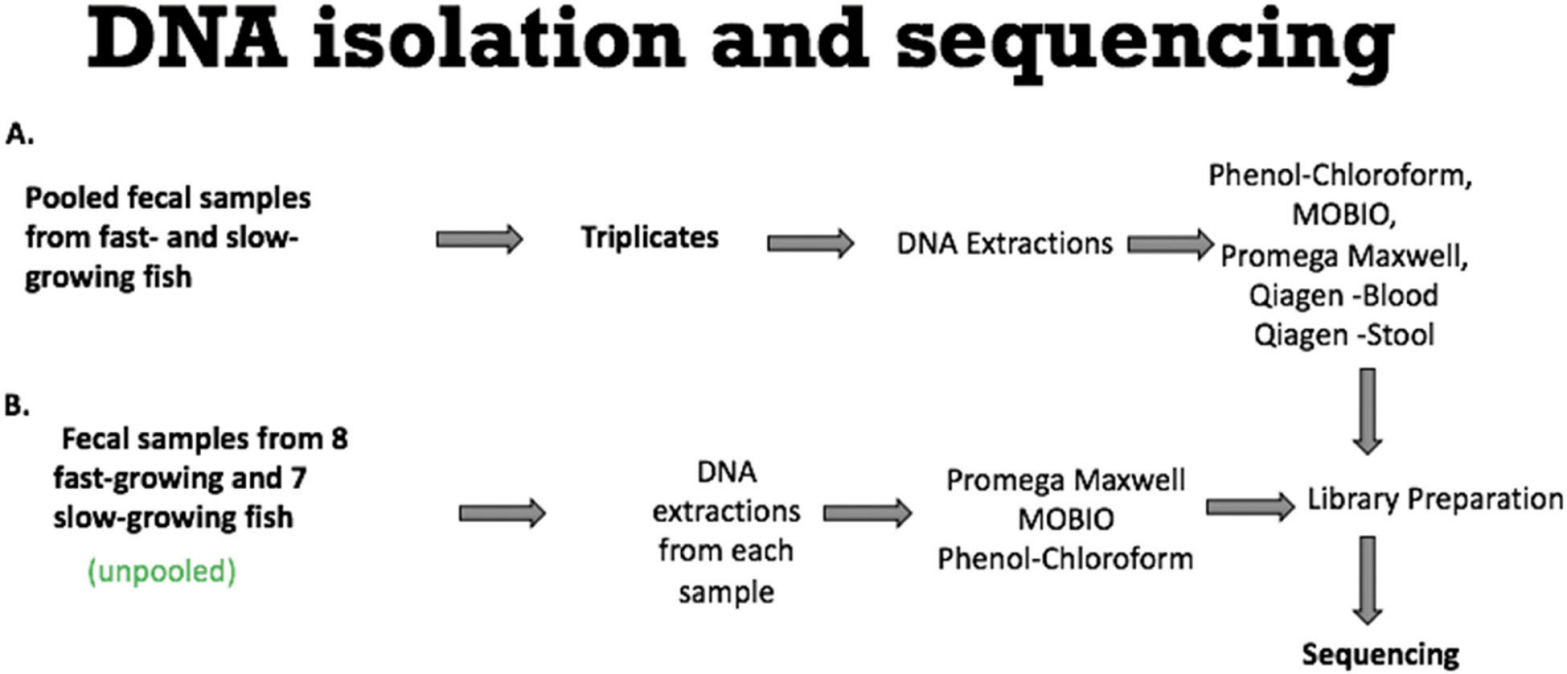
Experimental design for DNA isolation and sequencing. **a**) DNA extraction comparison using pooled fecal samples from all fast- and slow-growing fish. Three pooled fecal samples from all fast and slow-growing fish were subjected to five different DNA extraction comparisons. **b**) Analysis of fecal sample (unpooled) from 8 fast and 7 slow-growing fish to study the microbial assemblages

For comparison of extraction methods, fecal samples from 8 fast-growing and 7 slow-growing fish were pooled together and DNA extraction was done in triplicate using five different extraction methods including PowerSoil® DNA Isolation Kit **(**MO BIO Laboratories, Inc., West Carlsbad, CA), Promega Maxwell DNA Isolation Kit (Promega Corporation, Madison, WI), Qiagen Blood/Tissue, Qiagen Stool (Qiagen, Germantown, MD) and Phenol-chloroform (Phenol: Chloroform 5:1, SIGMA) extraction method [40]. The individual biological replicates DNA samples extracted using the MOBIO, Promega, and Phenol-chloroform methods were used for the analysis of the gut microbiota of fast-growing versus slow-growing trout. More detail of the DNA extraction methods is provided in Additional file 2 and steps of experimental design using pooled and unpooled samples have been included in Fig. 5. After extraction, DNA concentration was measured using Qubit (Qubit fluorometer, v3.11) (Invitrogen, Carlsbad, CA) and DNA was visualized by gel electrophoresis. All DNA extractions were stored at − 80 °C until library preparation.

Before library preparation, concentrations of all DNA samples were normalized to 2 ng/μL for PCR amplification using a Qubit fluorometer. The primers 515F and 926R (Integrated DNA Technologies) (EMP; http://www.earthmicrobiome.org/emp-standard-protocols/16s/) were used to target the 16S rRNA marker gene using polymerase chain reaction (PCR). The final PCR reaction consisted of 5 μL buffer, 1.5 μL 50 mM MgCl_2_, 2 μL 10 mM dNTP, 0.2 μL Taq polymerase, 3 μL Kb extender, 1 μL 10 μM primer, 5 μL DNA template and 7.3 μL nuclease-free water. PCR amplification and sample indexing (a total of 67 samples multiplexed) was performed [41]. The amplification conditions were 94 **°**C for 45 s, 50 **°**C for 60 s, 72 **°**C for 90 s for 35 cycles. Amplification was preceded by a 10-min preheating step at 94 **°**C and followed by a 10-min elongation step at 72 **°**C. Amplification of each sample was performed in triplicate and combined to a final volume of 75 μL. The indexed samples were then normalized (240 ng/reaction) and pooled for sample purification purposes. The pooled amplicon was purified using Promega PCR purification kit (Promega Corporation, Madison, WI) and visualized on a 1.5% agarose gel stained with ethidium bromide. A DNA fragment of the amplicon for each sample was excised from the DNA gel with a clean, sharp scalpel and collected in nuclease-free sterile tubes. QIAquick gel-extraction kit was used to purify DNA from the resulting gel slice (Qiagen, Germantown, MD) according to the manufacturer’s recommendation. The concentration of the gel-extracted library was assessed with a Qubit fluorometer (Invitrogen, Carlsbard, CA) and fragment size was determined using an Agilent 2100 Bioanalyzer (Agilent, Santa Clara, California). Final qPCR-based quantification of the library was done using a KAPPA quantification kit (Roche, Pleasanton, CA). Sequencing was done using 250 bp paired-end sequencing using a 300 cycle V2 reagent cartridge on an Illumina Miseq flow cell (Illumina, Inc., San Diego, CA) according to the manufacturer’s instructions (Miseq System Guide) [42]. The output file was demultiplexed and converted to fastq on the Illumina MiSeq (Illumina, Inc., San Diego, CA).

### Bioinformatics analyses

During sequencing, the adaptor trimming option was selected to remove the adaptors from the sequences. Samples were demultiplexed prior to using Mothur on the basis of Illumina Miseq instructions and total 8,500,662 paired-end raw sequences were obtained from Miseq Software (version 2.6.2.3). Sequencing data were analyzed using Mothur (v.1.40.2, www.mothur.org) according to the Mothur Illumina Miseq standard operating procedure (SOP) [43, 44] with several modifications. After forming contigs, the total number of sequences were 3,972,613 the median length (371 bp) of the sequences was determined. Sequences with ambiguous base pairs were removed by using the *screen*.*seqs* command, which ultimately reduced the sequences to 3,340,066. The *split.abund* command was used to keep sequences with more than two reads [45]. Since we were sequencing V4-V5 region, we customized our reference alignment using primer for V4-V5 region (http://blog.mothur.org/2016/07/07/Customization-for-your-region/), sequences were then trimmed on the basis of alignment start and end using *pcr.seqs* command. *Filter.seqs* command was used to filter the sequences with QC value> 25 and 3,314,628 sequences were then aligned to the SILVA v123 database and sequences that failed to align, or classified as Archaea, chloroplast, eukaryotic mitochondrial, or unknown sequences, were excluded from the analysis. Sequences detected by UCHIME as chimeric were removed from the analysis. The remaining sequences (3,150,919) were clustered using VSEARCH [46] at a threshold of > 97% sequence similarity. The *remove.rare* command was used to remove operational taxonomic units (OTUs) with abundance less than ten reads [47, 48]. Two samples (one fast-growing extracted using Promega Maxwell method and one slow-growing fish extracted using Phenol-chloroform method) were excluded from the analysis because sequences in these samples did not pass the quality control and filtering steps. The parameters and the commands used to analyze the data are included in Additional file 3.

### Statistical analysis

To study the effect of DNA extraction methods on microbial community profiling, Bray-Curtis distances were compared and nMDS ordination was used for visualization using Primer 7 (version 7.0.13) for windows ((PRIMER-E Ltd., Plymouth, UK). To test for a significant effect of extraction method, we used Permutational Multivariate Analysis of Variance (PERMANOVA) on the basis of Bray-Curtis dissimilarity matrices by considering extraction technique as a fixed effect and using type III sum of squares and unrestricted permutation of data with 999 permutations. SIMPROF (Similarity Profile) was performed to test the inter-sample variation on the replicate samples with a significant cut off value of 0.5 (95% similarity). Similarly, Beta diversity of fast-growing and slow-growing samples were calculated using Bray-Cutis dissimilarity matrices representing pairwise (sample to sample) distances to test the variation among fast and slow-growing fish. Non-metric multidimensional scaling ordination (nMDS) was used to explore the microbial communities in the fast-growing and slow-growing fish by considering the dissimilarity distance matrices among the samples. A one-way PERMANOVA was used to determine if the microbial assemblages differ with growth rate or fish breeding family, both considered as fixed effects. Moreover, alpha diversity was evaluated by comparing inverse Simpson diversity matrices for each group i.e. fish growth rate and fish families using R (R version 3.5.2).

To determine the microbial assemblages that are characteristics to the two growth rates and four families, an indicator species analysis was done in Mothur using *indicator* command [25, 49]. Taxa with indicator value greater than 40 and a (*p* < 0.05) were considered as significant indicators of fish growth rate or a breeding family [49]. All data files for the bioinformatics and statistical analyses are included in Additional files 3, 4, 5, 6, 7, 8 and 9_b.

The statistical significance of the rank body mass between the two groups was tested by a one-way Mann-Whitney U test with an alpha of p < 0.05 (Prism, GraphPad Software, Inc., La Jolla, CA).

## Supplementary information

**Additional file 1.** Taxa information of trout fecal samples. Taxonomy information of fecal samples using Promega Maxwell DNA extraction kit.

**Additional file 2.** DNA extraction protocol. DNA extraction Protocols of three different extraction techniques: PowerSoil® DNA Isolation Kit - MO BIO, Phenol-Chloroform and Promega Maxwell 16 FFPE Plus LEV DNA Purification Kit.

**Additional file 3.** Mothur SOP. Mothur Standard Operating procedure for microbiota profiling.

**Additional file 4.** Mothur Analysis log file. Logfile containing Mothur commands and results during Mothur analysis. Result of each step can be tracked in this file.

**Additional file 5.** Metadata file for fast and slow, Extraction technique . Detailed information regarding the fast and slow-growing fish samples (weight, length, sex, condition factor).

**Additional file 6.** _a and 6_b. Mothur shared file. a) Fast- and slow-growing fish fecal sample analysis shared file Extraction technique shared file. The data in the shared file contains the relative abundance of OTUs in multiple samples. This file can be used for further analysis, e.g. alpha and beta diversity measurements using different packages (R, Primer).

**Additional file 7.** Beta diversity analysis result file of the fast- and slow-growing fish using PRIMER. PRIMER was used for multivariate analysis. PERMANOVA results for fast and slow-growing fish analysis and PERMANOVA results for fish families.

**Additional file 8.** Beta diversity analysis result file of extraction technique using PRIME. PERMANOVA result for DNA extraction comparison.

**Additional file 9.** _a and 9_b. Mothur taxonomy file. a) Fast- and slow-growing fecal analysis taxonomy file Extraction techniques taxonomy file. Data in taxonomy file contains the taxa information of OTUs that matched with the Silva database.

## Data Availability

All data are provided in additional files.

## Abbreviations

nMDS: non-metric multidimensional scaling
OTUs: Operational Taxonomic units
PERMANOVA: Permutational Multivariate Analysis of Variance
rRNA: Ribosomal RNA
