# Supplementary material for: Analysis of the fecal microbiota of fast- and slow-growing rainbow trout (Oncorhynchus mykiss)
Source: BMC Genomics. 2019 Oct 29;20:788. doi: 10.1186/s12864-019-6175-2 (PMC6819385; doi:10.1186/s12864-019-6175-2)
Supplement: Supplementary file 2 — Additional file 2. DNA extraction protocol. DNA extraction Protocols of three different extraction techniques: PowerSoil® DNA Isolation Kit - MO BIO, Phenol-Chloroform and Promega Maxwell 16 FFPE Plus LEV DNA Purification Kit. [file 12864_2019_6175_MOESM2_ESM.docx]

**Additional file 2**

**PowerSoil® DNA Isolation Kit** - **MO BIO**

MO BIO extraction in particular includes bead beating steps to induce cell lysis. 250 mg of the sample was added to 700 μL lysis solution and 100 μL lysis additive. The sample was subjected to bead beater equipment then vortexed at maximum speed for 5 minutes. Samples were then centrifuged, 100 μL of binding solution was added to the supernatant, and the tube was incubated on ice for 5 minutes. The sample was then spun, and supernatant was transferred to the column. DNA bound to the column was then precipitated with ethanol and washed with wash solutions. Finally, the DNA was collected in 50ul of nuclease free water.

**Phenol-Chloroform**

Certain optimizations regarding the amount of samples, concentration of buffers and enzymes, temperature and the volume of phenol chloroform were made in the standard protocol to improve concentration and quality. Each fecal sample (0.25 g) was washed with 0.85% 2X PBS and spun (8000 rpm for 15 min) to a pellet. 0.5 ml 10mM Tris-HCL was added and sample were pre- treated with 10ul of 2.5mg/ml lysozyme, followed by water bath incubation for 1 hour at 37°C. Afterwards, 1 mL of lysis buffer (9.34 ml TE buffer pH 8, 0.6 mL 10% SDS, 60 μL of 20mg/mL proteinase K) was added and mixed in gently by tapping. The mixture incubated again in a water bath at 37 °C for 1-2 hours, inducing cell lysis. After incubation, an equal volume of phenol-chloroform (1:1) (700ul each) was added and mixed in gently until the mixture became turbid. The mixture was then centrifuged to 12000 g at 4 °C for 15 minutes, separating the mixture into three layers; the upper aqueous phase was transferred to a sterile microcentrifuge tube. The phenol-chloroform step was repeated to remove the protein. DNA was precipitated by adding 2-3 volumes of pure ethanol, incubating overnight, and spinning at 14000 g was done for 15min at 4 °C. The DNA pellet was rinsed with 70% ethanol and spun at 14000 g for 5 min. After centrifugation, the pellet was dried under a laminar flow hood for half an hour and solubilized in nuclease free water.

**Promega Maxwell 16 FFPE Plus LEV DNA Purification Kit**

Extractions were performed according to the manufacturer’s recommendation. However, we added 20 μL of lysozyme (not mentioned in protocol) to facilitate the lysis of cell walls of the Gram-positive bacteria. A miniscule amount of fecal sample was added to a microtube containing 160 μL of lysis buffer, 20 μL proteinase k solution, and 20 μL lysozyme. The mixture was incubated at 70 **°**C overnight, and after incubation, 400 μL of lysis buffer was added to the mixture and the sample was vortexed briefly. The samples were then subjected to the Maxwell 16 Automated DNA purification machine and the DNA was collected in a 50ul elution buffer.

**Qiagen-Blood/Tissue Kit**

DNA extraction was done according to manufacturer’s recommendations.

**Qiagen Stool kit**

DNA extraction was done according to manufacturer’s recommendations.
